# Development of a sustainable diet index in US adults

**DOI:** 10.1186/s12937-024-00943-3

**Published:** 2024-04-10

**Authors:** Sukyoung Jung, Heather A. Young, Barbara H. Braffett, Samuel J. Simmens, Cynthia L. Ogden

**Affiliations:** 1 Department of Epidemiology, Milken Institute School of Public Health, The George Washington University, Washington, DC USA; 2 The Korean Institute of Nutrition, Hallym University, 1 Hallymdaehak-gil, Chuncheon, 24252 South Korea; 3 Department of Biostatistics and Bioinformatics, Milken Institute School of Public Health, The George Washington University, Washington, DC USA

**Keywords:** Sustainable diets, Diet index, Multidimensional assessment, Environmental impact, Dietary patterns

## Abstract

**Background:**

A transformation towards healthy diets through a sustainable food system is essential to enhance both human and planet health. Development of a valid, multidimensional, quantitative index of a sustainable diet would allow monitoring progress in the US population. We evaluated the content and construct validity of a sustainable diet index for US adults (SDI-US) based on data collected at the individual level.

**Methods:**

The SDI-US, adapted from the SDI validated in the French population, was developed using data on US adults aged 20 years and older from the National Health and Nutrition Examination Survey, 2007–2018 (*n* = 25,543). The index consisted of 4 sub-indices, made up of 12 indicators, corresponding to 4 dimensions of sustainable diets (nutritional quality, environmental impacts, affordability (economic), and ready-made product use behaviors (sociocultural)). A higher SDI-US score indicates greater alignment with sustainable diets (range: 4–20). Validation analyses were performed, including the assessment of the relevance of each indicator, correlations between individual indicators, sub-indices, and total SDI-US, differences in scores between sociodemographic subgroups, and associations with selected food groups in dietary guidelines, the alternative Mediterranean diet (aMed) score, and the EAT-Lancet diet score.

**Results:**

Total SDI-US mean was 13.1 (standard error 0.04). The correlation between SDI-US and sub-indices ranged from 0.39 for the environmental sub-index to 0.61 for the economic sub-index (Pearson Correlation coefficient). The correlation between a modified SDI-US after removing each sub-index and the SDI-US ranged from 0.83 to 0.93. aMed scores and EAT-Lancet diet scores were significantly higher among adults in the highest SDI-US quintile compared to the lowest quintile (aMed: 4.6 vs. 3.2; EAT-Lancet diet score: 9.9 vs. 8.7 *p* < .0001 for both).

**Conclusions:**

Overall, content and construct validity of the SDI-US were acceptable. The SDI-US reflected the key features of sustainable diets by integrating four sub-indices, comparable to the SDI-France. The SDI-US can be used to assess alignment with sustainable diets in the US. Continued monitoring of US adults’ diets using the SDI-US could help improve dietary sustainability.

**Supplementary Information:**

The online version contains supplementary material available at 10.1186/s12937-024-00943-3.

## Introduction

The Food and Agriculture Organization of the United Nations (FAO) defines sustainable diets as ‘diets with low environmental impacts which contribute to food and nutritional security and to healthy life for present and future generations’ and ‘are protective and respectful of biodiversity and ecosystems, culturally acceptable, accessible, economically fair and affordable; nutritionally adequate, safe, and healthy; while optimizing natural and human resources’ [1]. Recent studies highlight the need to transform the current food system by changing individual dietary practices along with technological and organizational innovations to achieve sustainability [2–4].

Given the FAO definition, sustainable dietary patterns should be assessed using a multidimensional approach with holistic consideration of four dimensions of diet (nutritional, environmental, economic, and sociocultural dimensions). There have been attempts to operationalize multidimensional sustainable diets. A sustainable diet index (SDI) consisting of 4 sub-indices with 7 indicators was developed and validated using a French cohort [5], and the SDI for French adults (SDI-France) was inversely associated with the incidence of obesity [6], cancers [7], and cardiovascular diseases [7]. A SDI consisting of 3 sub-indices with 6 indicators was developed using a Spanish cohort, and the SDI for Spanish adults (SDI-Spain) was inversely associated with all-cause and cardiovascular mortality [8].

To date, there has been no attempt to comprehensively assess alignment with sustainable diets among US adults. We therefore constructed and evaluated an SDI for US adults (SDI-US) based on the prior scoring system that was developed for French adults [5] using data from 6 consecutive cycles of the National Health and Nutrition Examination Survey (NHANES).

## Methods

### Study population

NHANES, an ongoing, cross-sectional, and nationally representative survey, was established to assess the health and nutritional status of the non-institutionalized civilian population in the US [9]. Since 1999, NHANES has continuously collected data and publicly released it every 2-years. To ensure the representativeness of the non-institutionalized civilian US population, NHANES uses a complex and multistage probability sampling design [10]. NHANES data collection is conducted throughout the year and includes a household interview, a mobile examination center (MEC) visit (standardized physical examinations, laboratory tests, health interviews, and dietary assessments including 24-hour dietary recalls conducted by trained staff), and post-MEC follow-up [11].

Among 34,770 adults aged 20 years and older, we excluded participants who were pregnant or breastfeeding (*n* = 580); did not complete the first 24-hour dietary recall (*n* = 3,944); or were missing information on serum 25(OH)D level (*n* = 2,058), the components for the SDI-US score calculation (family income, food expenditures, ready-made product use behaviors) (*n* = 2,628), or education level (*n* = 17). The final analytic sample included 25,543 adults (Supplementary Fig. 1).

### Data collection

During the household interview, information on demographics (e.g., age, sex, race and Hispanic origin, the highest level of school or the highest degree received, annual family income, and household size), food expenditures at the family level, and ready-made product use behaviors were collected.

During the MEC examination, a 24-hour dietary recall interview was conducted along with the collection of biological specimens, including blood. Trained dieticians conducted the 24-hour dietary recall interview to collect detailed dietary information from survey participants. The 24-hour dietary recall interview included information on the description, quantity, time and place of eating of all foods, beverages, and water consumed during the past 24-hours (from midnight to midnight). Respondents reported amounts of foods and beverages consumed with the assistance of a standard set of measuring guides such as glasses, bowls, mugs, bottles, household spoons, and measuring tools. The Automated Multiple Pass Method was administered to obtain complete and accurate food recall [12]. Energy, nutrients, and food components of all foods and beverages reported in NHANES 2007–2018 were obtained from the United States Department of Agriculture (USDA)’s Food and Nutrient Database for Dietary Studies 4.1, 5.0, 2011–2012, 2013–2014, 2015–2016, and 2017–2018 [13].

To account for vitamin D intake from both food and skin synthesis, serum total 25(OH)D (nmol/L) was used in this study. Serum total 25(OH)D was defined as the sum of 25(OH)D3 and 25(OH)D2, which were measured using a fully validated standardized liquid chromatography-tandem mass spectrometry method [14].

### Sustainable diet index-US

The SDI-US score was adapted from the SDI-France developed by Seconda et al. [5], applying the same rationale for indicator selection (Table 1). Seconda et al. used information from several review studies to select and define indicators of diet sustainability [5]. Similarly, for the SDI-US, indicators were selected if (1) they covered at least one of the four dimensions of diet (nutritional, environmental, economic, and sociocultural) and (2) could be assessed at an individual level [5].

**Table 1:** Description of selected indicators and calculation of the total sustainable diet index-US and sub-index scores

				SDI-US	
Sub-index	Measure^a^	Relationship with sustainable diets^a^	Indicators in the sub-index	Points allocating	Assessment
Nutritional (/5)	Dietary diversity index	Diet diversity with nutrient adequacy is essential to avoid malnutrition and negative health outcomes.	1–1) Nutrient-Rich Foods9.3 Index^b^	1-point: ind ≤ 4.1 2-point: 4.1 < ind ≤ 10.6 3-point: 10.6 < ind ≤ 18.2 4-point: 18.2 < ind ≤ 30.5 5-point: ind > 30.5	Nutrition sub-index= the sum of points from indicator 1–1 to 1–2 × 1/2
	Micronutrient (vitamins and minerals) deficiencies		1–2) Mean Nutrient Adequacy Ratio^c^	1-point: ind ≤ 60.2 2-point: 60.2 < ind ≤ 68.1 3-point: 68.1 < ind ≤ 74.2 4-point: 74.2 < ind ≤ 80.5 5-point: ind > 80.5	
Environmental (/5)	Water footprint	Clean water resource is becoming scarce in zones.	2 − 1) Freshwater withdrawals (L) per serving food^d^	1-point: ind > 549.9 2-point: 377.1 < ind ≤ 549.9 3-point: 263.7 < ind ≤ 377.1 4-point: 161.5 < ind ≤ 263.7 5-point: ind ≤ 161.4	Environment sub-index= the sum of points from indicator 2 − 1 to 2–6 × 1/6
			2–2) Stress-weighted water use (L) per serving food^d^	1-point: ind > 18,475 2-point: 12,806 < ind ≤ 18,475 3-point: 9079 < ind ≤ 12,806 4-point: 5601 < ind ≤ 9079 5-point: ind ≤ 5601	
	Nitrogen footprint	Nitrogen balance is essential to avoid eutrophication and harmful algae blooms.	2–3) Acidifying emissions (g SO_2_eq, CML2 baseline) per serving food^d^	1-point: ind > 34.4 2-point: 22.6 < ind ≤ 34.4 3-point: 15.4 < ind ≤ 22.6 4-point: 9.3 < ind ≤ 15.4 5-point: ind ≤ 9.3	
			2–4) Eutrophying emissions (g PO_4_^3−^eq, CML2 Baseline) per serving food^d^	1-point: ind > 28.0 2-point: 16.3 < ind ≤ 28.0 3-point: 10.2 < ind ≤ 16.3 4-point: 6.1 < ind ≤ 10.2 5-point: ind ≤ 6.1	
	Carbon footprint	Anthropogenic greenhouse gas emissions contribute to climate change.	2–5) Greenhouse gas emissions (kg CO_2_eq, IPCC 2013 includes feedbacks) per serving food^d^	1-point: ind > 5.8 2-point: 3.4 < ind ≤ 5.8 3-point: 2.2 < ind ≤ 3.4 4-point: 1.4 < ind ≤ 2.2 5-point: ind ≤ 1.4	
	Land use	The availability of arable land is limited; moreover, land use change impacts the biodiversity preservation.	2–6) Land use (m^2^) per serving food^d^	1-point: ind > 13.0 2-point: 5.9 < ind ≤ 13.0 3-point: 3.7 < ind ≤ 5.9 4-point: 2.1 < ind ≤ 3.7 5-point: ind ≤ 2.1	
Economic (/5)	Affordability	Healthy diet should be available at affordable prices to all, specifically to low-income consumers.	3) Proportion of income devoted to diet	1-point: ind > 34.1 2-point: 20.0 < ind ≤ 34.1 3-point: 13.3 < ind ≤ 20.0 4-point: 9.0 < ind ≤ 13.3 5-point: ind ≤ 9.0	Economic sub-index= the sum of points × 1
Sociocultural (/5)	Ready-made products	The use of ready-made products minimizes cooking activities and thus limit the opportunity for social exchange, cultural heritage preservation and trying diverse recipes.	4 − 1) Frequency of meals not home prepared and from a fast-food or pizza place	1-point: ind > 4 2-point: 2 < ind ≤ 4 3-point: 1 < ind ≤ 2 4-point: 0 < ind ≤ 1 5-point: ind = 0	Sociocultural sub-index= the sum of points from indicator 4 − 1 to 4 − 3 × 1/3
			4 − 2) Frequency of ready-to-eat products	1-point: ind > 5 2-point: 3 < ind ≤ 5 3-point: 1 < ind ≤ 3 4-point: 0 < ind ≤ 1 5-point: ind = 0	
			4 − 3) Frequency of frozen meals/pizza	1-point: ind > 7 2-point: 3 < ind ≤ 7 3-point: 2 < ind ≤ 3 4-point: 0 < ind ≤ 2 5-point: ind = 0	
**Total SDI-US = nutritional + environmental + economic + sociocultural (range: 4–20)**					

*Abbreviation* SDI-US, sustainable diet index-US; ind, indicator

^a^Source: Seconda L, Baudry J, Pointereau P, et al. Development and validation of an individual sustainable diet index in the NutriNet-Santé study cohort. *Br J Nutr* 2019;121:1166-77

^b^Sources: Fulgoni VL, 3rd, Keast DR, Drewnowski A. Development and validation of the nutrient-rich foods index: a tool to measure nutritional quality of foods; Drewnowski A. Defining nutrient density: development and validation of the nutrient rich foods index. *J Am Coll Nutr* 2009;28:421s–6s

^c^Source: Guthrie HA, Scheer JC. Nutritional adequacy of self-selected diets that satisfy the four food groups guide. *J Nutr Education* 1981;13:46 − 9

^d^Source: Poore J, Nemecek T. 2018. Reducing food’s environmental impacts through producers and consumers. Science 360:987–992 and Bryan T, Hicks A, Barrett B, et al. An environmental impact calculator for 24-h diet recalls. *Sustainability* 2019;11(23):6866

The SDI-US consists of 4 sub-indices that include 12 indicators for each NHANES participant. The nutritional sub-index includes 2 indicators: the Nutrient-Rich Foods (NRF) 9.3 index capturing dietary diversity and mean nutrient adequacy ratio (MAR) reflecting micronutrient deficiencies. The environmental sub-index includes 6 indicators: freshwater withdrawals and stress-weighted water use to represent the water footprint, acidifying emissions and eutrophying emissions to represent the nitrogen footprint, greenhouse gas emissions (GHGE) to represent the carbon footprint, and land use. The economic sub-index includes 1 indicator: the proportion of monthly income spent on food reflecting affordability. The sociocultural sub-index includes 3 indicators: frequency of meals not home prepared and from a fast-food or pizza place, frequency of ready-to-eat products, frequency of frozen meals/pizza reflecting ready-made food use behaviors (rather than assessment of ultra-processed food consumption as comparable to the SDI-France).

#### Nutritional indicators

To calculate the two nutritional indicators, we used dietary intake data from the MEC 24-hour dietary recall interview. First, the NRF9.3 index was calculated by summing the percent daily values (DV) for nine nutrients to encourage (protein, fiber, vitamins A, C, and E, calcium, iron, magnesium, and potassium) and subtracting the percent DV for three nutrients or food component to limit (saturated fat, added sugar, and sodium) based on nutrient recommendations from the Dietary Guidelines for Americans (DGAs) [15, 16]. Second, MAR was calculated as the average of the nutrient adequacy ratios (NARs) of 12 nutrients selected because 10% or more of US adults did not meet intake of these nutrient recommendations in 2015–2018. The nutrients included were: vitamin A (µg RAE), thiamin (mg), vitamin B_6_ (mg), folate (µg DFE), vitamin C (mg), vitamin E as alpha-tocopherol (mg), calcium (mg), magnesium (mg), iron (mg), zinc (mg), copper (mg) [17], and vitamin D as serum 25(OH)D (nmol/L). All nutrients for the NAR calculation except for serum 25(OH)D were adjusted for total energy intake using a residual method [18] to adjust for measurement error due to underreporting [19–21] and to reduce variation in intake due to age, sex, body size, and metabolic efficiency [22]. The NAR was calculated as the ratio of the level of those nutrients consumed to recommended dietary allowance [23] or the National Academy of Medicine-recommended cut-point for risk of deficiency of 25(OH)D (< 50 nmol/L) [24]: actual consumption of a selected nutrient / the recommended level of a selected nutrient.

#### Environmental indicators

To calculate the 6 environmental indicators, we used a predeveloped environmental impact calculator [25], which is based on a meta-analysis of 1,530 publications that allows us to assess the environmental impact of 43 food groups [freshwater withdrawal (L), stress-weighted freshwater withdrawal (L), acidifying emissions (g SO_2_eq), eutrophication emissions (g PO_4_^3−^eq), GHGE (kg CO_2_eq), and land use (m^2^)] [26]. The system boundaries were agricultural production, processing, packaging, and retail [26]. Each food reported in the 24-hour dietary recalls in NHANES 2007–2008 through 2017–2018 was hand-coded and matched to the 43 food groups in the calculator, and the respective environmental impacts were estimated.

#### Economic indicator

To assess the 1 economic indicator, we used responses to annual family income and the following questions: (1) “During the past 30 days, how much money did you spend at supermarkets or grocery stores?”; (2) “About how much money did you spend on food at these types of stores?”; (3) “During the past 30 days, how much money did you spend on eating out?”; (4) “During the past 30 days, how much money did you spend on food carried out or delivered?”. Total monthly food expenditures were calculated as the sum of the dollars spent using the above specific questions and annualized by multiplying by 12. Since total annual family income is available as a categorical variable, median values of each income range were used as a proxy for total family income. Finally, the proportion of income spent on food items was calculated as total annual food expenditures divided by total annual family income and expressed as a percentage.

#### Sociocultural indicators

To assess the 3 sociocultural indicators, we used responses to the following questions: (1) “During the past 7 days, how many meals did you get that were prepared away from home in places such as restaurants, fast food places, food stands, grocery stores, or from vending machines?” and “How many of those meals did you get from a fast-food or pizza place?” (valid range: 0–21); (2) “During the past 30 days, how often did you eat “ready to eat” foods from the grocery store?” (valid range: 0–90); (3) “During the past 30 days, how often did you eat frozen meals or frozen pizzas?” (valid range: 0–90). All indicator variables were continuous.

#### Scoring and calculation of the total sustainable diet index-US

The FAO definition of sustainable diets equally weights the nutritional, environmental, economic, and sociocultural dimensions in terms of importance [1]. In addition, to make it comparable to other countries (e.g., SDI-France), equal weighting was applied to all four sub-indices. For the scoring, all indicator values, except for the sociocultural indicators, were categorized into quintiles and scored from 1 to 5, where a higher score indicated better nutritional quality, less damage to the environment, and better affordability. For the sociocultural indicators, we assigned a score of 5 to participants who reported zero frequency to the questions. For all other participants, we assigned a score of 4 to those in the first quartile of the indicator and a score of 1 to those in the fourth quartile of the indicator. A total score for each sub-index was calculated by taking the average of all indicator points within the sub-index. Thus, the SDI-US ranged from 4 to 20, with a higher score indicating a greater alignment with a sustainable dietary pattern. The details of the calculation of the SDI-US are presented in Table 1.

### Statistical analysis

As described in Table 2, content validity was evaluated using the same strategies as in the SDI-France development study [5]: (1) the relevance of each indicator was described in terms of the FAO definition of sustainable diets; (2) the associations between the individual indicators and the total SDI-US score were examined to test which indicators have the most influence on the total SDI-US score; (3) the correlations between the sub-indices or modified SDI-US where each sub-index was removed (e.g., SDI-US without the nutritional sub-index) and the total SDI-US score were estimated to determine whether all four sub-indices have a balanced influence on the total SDI-US.

**Table 2:** Strategies used to evaluate the validity of the sustainable diet index-US (SDI-US)

Question	Strategy	Output
**Content validity**		
1. Does the index capture the key dimensions of sustainable diets defined by the Food and Agriculture Organization?	Followed and checked the relevance of each indicator based on the original SDI development study (Seconda et al., *Br J Nutr* 2019;121:1166-77)	Table 1
2. Does a single individual indicator have an oversized impact on the total score?	Estimated Pearson correlations between the individual indicators and the total SDI-US	Table 3
3. Does one sub-index have an oversized impact on the total score?	Estimated Pearson correlations between the sub-indices and the total SDI-US and between the index after removing each sub-index and the total SDI-US	Table 3
**Construct validity**		
4. Does the index differentiate between groups with known differences in diet quality? i.e., does it have concurrent-criterion validity?	Applied general linear models to compare male and female, older and younger adults, different race/Hispanic origins, and different education level groups	Table 4
5. Does the index correctly measure what it should measure (sustainability of diets)? e.g., does plant-based foods consumption increase as the SDI-US score increases?	Evaluated the association between the SDI-US and the consumption of selected food groups included in the DGA, the alternative Mediterranean diet score, and the EAT-Lancet diet score	Table 5

Construct validity of SDI-US was evaluated in 2 ways. First, sociodemographic characteristic differences in scores were compared using linear regression models to test whether the index differentiated between groups with known differences in diet quality (concurrent-criterion validity). Second, the associations of SDI-US with DGA recommendations for select food groups, the alternative Mediterranean diet score (aMed) [27], and the EAT-Lancet diet score [28] were examined using linear regression models to assess if there were positive associations between SDI-US and other dietary sustainability indices (e.g., does plant-based food consumption increase as the SDI-US score increases?). The DGA food groups included: total vegetables (dark green vegetables, red and orange vegetables, legumes, starchy vegetables, other vegetables), fruits, grains (whole grains, refined grains), dairy, protein foods (meats, poultry, eggs, seafood, nuts, seeds, soy products) [29, 30]. The aMed (range: 0–9) was calculated using the consumption of vegetables, legumes, fruits, whole grains, nuts, fish, ratio of monosaturated fatty acids to saturated fatty acids, red and processed meat, and alcohol; a higher score indicated a closer resemblance to the Mediterranean diet [27]. The EAT-Lancet diet score (range: 0–14) was based on 14 dietary recommendations related to consumption of (1) rice, wheat, corn and other, (2) tubers and starchy vegetables, (3) all vegetables, (4) all fruits, (5) dairy products, (6) beef, lamb, pork, (7) chicken, other poulty, (8) eggs, (9) fish, (10) beans, lentils, peas, 11) soy foods, 12) peanuts or tree nuts, 13) added fats (ratio of 0.8 for unsaturated to saturated fat intake), and 14) added sugars [28]. To address the differences in units, we used conversion factors to translate serving equivalents to grams [31]. For each component, 1 point was assigned if a criteria were met and 0 points otherwise, and then all points from each component were summed.

We conducted sensitivity analyses to determine whether various modified ways of constructing the SDI would lead to similar quantile assignments as the SDI-US. Modifications tests were as follows: (1) using the Healthy Eating Index (HEI)-2015 instead of the NRF9.3 to account for the possibility that better nutritional composition does not necessarily translate into an overall healthier diet; (2) using 5 environmental indicators after excluding one water-related indicator (freshwater use) to consider the possible effect of double counting on water in relation to the other indicators; (3) by additionally including food security level and food price level (assessed by the Purchase to Plate National Average Prices for NHANES 2011–2018) [32] to the economic sub-index to better represent food affordability; (4) we calculated the SDI-US by additionally including eating together with family or friends to the sociocultural sub-index because higher frequency of ready-to-eat meals may not necessarily indicate negative sociocultural practices related to social exchange or trying diverse recipes if people are eating together. However, this was tested only in NHANES 2007–2010 due to data availability. For the cross-classification analyses, we considered the performance was as “good” if more than 50% of the participants were correctly classified and less than 10% were misclassified, while the performance was considered “poor” if less than 50% of the participants were correctly classified and more than 10% were misclassified [33]. For a kappa value, ≥ 0.61 indicated a good agreement, 0.41–0.60 indicated an acceptable agreement, 0.21–0.40 indicated a fair agreement, and < 0.20 indicated a poor agreement [33].

Dietary Day 1 sample weights and survey design variables (strata and primary sampling units) were used in all analyses to adjust for unequal probabilities of selection due to the complex sampling design, noncoverage, nonresponse, and day of the week for the 24-hour dietary recall. Statistical analyses were conducted using SAS software (version 9.4, SAS Institute, Cary, NC, USA) with survey analysis procedures, and an α level of 0.05 was considered statistically significant.

## Results

From 2007 to 2018, the proportion of older adults (aged ≥ 60 years) increased from 24.6 to 29.7%. The proportion of non-Hispanic white adults decreased from 72.5 to 64.2%. The proportion of adults with at least some college education increased from 55.4 to 61.0%. The proportion of single-person households decreased from 14.1 to 12.9% (Supplemental Table 2).

During 2007–2018, the overall mean total SDI-US was 13.1 points (standard error (SE) 0.04) (Table 3). Mean SDI-US in quintile 1 was 9.5 (SE 0.02) and 16.5 (SE 0.02) in quintile 5.

**Table 3:** Weighted mean and standard error of total sustainable diet index-US (SDI-US), sub-index, and individual indicator scores by sustainable diet index-US quintile, US adults, NHANES 2007–2018

	Q1 (*n* = 5115)	Q2 (*n* = 5015)	Q3 (*n* = 5181)	Q4 (*n* = 5125)	Q5 (*n* = 5107)	Total (*n* = 25,543)	Correlation coefficient^b^
Median total SDI-US score (min, max)	9.7 (4.3, 10.8)	11.7 (11.0, 12.5)	13.0 (12.5, 13.7)	14.4 (13.8, 15.2)	16.1 (15.3, 20.0)	13.1 (4.3, 20.0)	NA
	*Weighted mean (standard error)* ^*a*^						
Total SDI-US score	9.5 ± 0.02	11.7 ± 0.01	13.1 ± 0.01	14.5 ± 0.01	16.5 ± 0.02	13.1 ± 0.04	1.00
**Sub-Index Scores & Indicator Values**							
Nutritional	2.1 ± 0.03	2.6 ± 0.03	3.0 ± 0.03	3.4 ± 0.03	4.0 ± 0.02	3.1 ± 0.02	0.54^**^
Nutrient Rich Foods 9.3 index score	5.8 ± 0.3	11.2 ± 0.4	16.7 ± 0.5	22.0 ± 0.5	32.9 ± 0.6	18.1 ± 0.3	0.42^**^
Mean nutrient adequacy ratio	63.4 ± 0.3	67.7 ± 0.3	70.9 ± 0.3	73.9 ± 0.3	77.9 ± 0.2	71.0 ± 0.2	0.43^**^
Environmental	2.2 ± 0.03	2.6 ± 0.03	2.9 ± 0.03	3.1 ± 0.04	3.6 ± 0.02	2.9 ± 0.01	0.39^**^
Fresh water use (L)	518 ± 8	444 ± 10	387 ± 8	337 ± 9	242 ± 5	382 ± 4	-0.27^**^
Stress-weighted water use (L)	17,275 ± 288	14,735 ± 326	13,244 ± 273	11,637 ± 309	8676 ± 175	13,004 ± 144	-0.26^**^
Acidifying emissions (g SO_2_eq)	37.5 ± 0.6	28.8 ± 0.7	22.1 ± 0.4	18.6 ± 0.5	12.0 ± 0.3	23.5 ± 0.3	-0.38^**^
Eutrophying emissions (g PO_4_^3−^eq)	29.9 ± 0.5	23.2 ± 0.6	17.0 ± 0.4	14.3 ± 0.5	8.7 ± 0.2	18.3 ± 0.2	-0.34^**^
Greenhouse gas emissions (kg CO_2_eq)	7.0 ± 0.2	5.4 ± 0.2	3.9 ± 0.1	3.2 ± 0.1	2.0 ± 0.1	4.2 ± 0.1	-0.32^**^
Land use (m^2^)	17.4 ± 0.5	13.0 ± 0.5	8.7 ± 0.3	7.0 ± 0.3	3.7 ± 0.1	9.8 ± 0.2	-0.28^**^
Economic	2.0 ± 0.03	2.7 ± 0.03	3.2 ± 0.03	3.7 ± 0.03	4.3 ± 0.02	3.2 ± 0.02	0.61^**^
Share of budget to food (%)	47.7 ± 1.5	30.4 ± 1.0	22.7 ± 0.9	17.4 ± 0.9	10.0 ± 0.2	25.1 ± 0.6	-0.27^**^
Sociocultural	3.3 ± 0.02	3.8 ± 0.02	4.0 ± 0.02	4.2 ± 0.02	4.5 ± 0.01	4.0 ± 0.01	0.47^**^
Frequency of meals from a fast-food or pizza place (times during the last 7 days)	3.1 ± 0.1	2.0 ± 0.1	1.6 ± 0.1	1.1 ± 0.1	0.8 ± 0.04	1.7 ± 0.03	-0.32^**^
Frequency of ready-to-eat products (times during the last 30 days)	3.6 ± 0.1	2.5 ± 0.2	1.9 ± 0.1	1.3 ± 0.1	0.8 ± 0.1	2.0 ± 0.1	-0.18^**^
Frequency of frozen meals and pizza (times during the last 30 days)	5.1 ± 0.2	2.9 ± 0.1	2.6 ± 0.2	1.9 ± 0.1	1.3 ± 0.1	2.7 ± 0.1	-0.19^**^
**Index scores after removing each sub-index**							
SDI-US without nutritional sub-index	7.5 ± 0.03	9.1 ± 0.03	10.1 ± 0.03	11.0 ± 0.03	12.4 ± 0.02	10.1 ± 0.02	0.85^**^
SDI-US without environmental sub-index	7.3 ± 0.03	9.1 ± 0.03	10.2 ± 0.03	11.3 ± 0.04	12.8 ± 0.03	10.2 ± 0.04	0.85^**^
SDI-US without economic sub-index	7.6 ± 0.02	9.0 ± 0.03	9.9 ± 0.03	10.8 ± 0.03	12.1 ± 0.03	9.9 ± 0.03	0.83^**^
SDI-US without sociocultural sub-index	6.3 ± 0.03	7.9 ± 0.02	9.1 ± 0.02	10.2 ± 0.02	12.0 ± 0.02	9.2 ± 0.03	0.93^**^

*Abbreviations* NHANES, National Health and Nutrition Examination Survey; Q, quintile; SDI-US, sustainable diet index-US

^a^Estimated mean and standard error were obtained using the linear regression model and weighted (dietary Day 1 sample weights)

^b^The Pearson correlation coefficients of the individual indicators, the sub-indices, and the index after removing each sub-index with the total SDI-US were estimated

^**^P value < 0.0001

### Validation

Table 2 shows strategies used to evaluate the validity of SDI-US.

#### Content validity

Question-1. Does the index capture the key dimensions of sustainable diets defined by the FAO?

The relevance of each indicator in the SDI-US is shown in Table 1 and shows that the key features (e.g., dietary diversity, adequate vitamins and minerals intake, lower environmental footprints, affordability, and reduced use of ready-made products) related to sustainable diets are captured by the index as comparable to the SDI-France [5].

Question-2. Does a single individual indicator have an oversized impact on the total score?

Table 3 shows weighted means and standard errors of the total SDI-US, sub-index, and individual indicator scores by SDI-US quintile. Overall mean SDI-US was 13.1 points (SE: 0.04). Mean SDI-US in the first quintile was 9.5 and increased to 16.5 in quintile 5. Nutritional indicators values increased by SDI-US quintile, while environmental, economic, and sociocultural indicators values decreased by SDI-US quintile. The correlation coefficients between each indicator and the total SDI-US ranged from − 0.18 (frequency of ready-to-eat products) to 0.43 (MAR). These results indicate that all correlation coefficients between individual indicators and SDI-US were similar (low to moderate), and no single indicator had an oversized impact on the total score.

Question-3. Does one sub-index have an oversized impact on the total score?

The correlations between each sub-index and the total SDI-US were moderate, ranging from 0.39 (environmental sub-index) to 0.61 (economic sub-index). These results indicate that no single sub-index had an excessive impact on the total score; correlation coefficients were similar (moderate) between sub-indices and the total SDI-US (Table 3). The correlation between the modified index after removing each sub-index and the total SDI-US were high, ranging from 0.83 (economic sub-index) to 0.93 (sociocultural sub-index). These results indicate that each sub-index has similar (high) impact on the total SDI-US (Table 3).

#### Construct validity

Question-4. Does the index differentiate between groups with known differences in diet quality (concurrent-criterion validity)?

The results of an evaluation of construct validity are shown in Tables 4 and 5. The SDI-US scores were higher among adults 60 + years compared to adults 20–39 years (13.9 vs. 12.6, *p* < .0001), females compared to males (13.4 vs. 12.9, *p* < .0001), non-Hispanic white adults compared to other groups (13.2 vs. 13.0, *p* = .0004), and among adults with a college degree or more education compared to those with less education (13.4 vs. 12.7, *p* < .0001) (Table 4). These results indicate that the SDI-US was significantly different across demographic subgroups with known differences in diet quality [34].

**Table 4:** Estimated mean and standard error of total sustainable diet index-US, sub-index, and individual indicator scores, by demographic factors, US adults, NHANES 2007–2018

	Weighted mean (standard error)^a^							
	Age 20–39	Age 60+	Male	Female	Hispanic, non-Hispanic black, & other	Non-Hispanic white	Less than college graduate	Some college or above
Total SDI-US score	12.6 ± 0.05	13.9 ± 0.04^b^	12.9 ± 0.04	13.4 ± 0.04^b^	13.0 ± 0.05	13.2 ± 0.04^b^	12.7 ± 0.05	13.4 ± 0.04^b^
**Sub-Index & indicator values**								
Nutritional	2.9 ± 0.03	3.4 ± 0.02^b^	3.1 ± 0.02	3.0 ± 0.03^b^	3.0 ± 0.02	3.1 ± 0.03^b^	2.8 ± 0.02	3.2 ± 0.02^b^
NRF9.3 index score	15.3 ± 0.4	22.2 ± 0.4^b^	16.3 ± 0.3	19.7 ± 0.4^b^	18.4 ± 0.3	17.9 ± 0.4	15.6 ± 0.4	19.5 ± 0.3^b^
MAR	69.3 ± 0.3	73.3 ± 0.2^b^	73.1 ± 0.2	68.9 ± 0.3^b^	68.9 ± 0.2	71.9 ± 0.3^b^	68.2 ± 0.2	72.6 ± 0.2^b^
Environmental	3.0 ± 0.02	2.9 ± 0.02^b^	2.7 ± 0.02	3.1 ± 0.02^b^	3.1 ± 0.02	2.8 ± 0.02^b^	3.0 ± 0.02	2.9 ± 0.02^b^
Fresh water use (L)	374 ± 7	384 ± 5	437 ± 5	329 ± 4^b^	347 ± 4	398 ± 5^b^	351 ± 5	401 ± 5^b^
Stress-weighted water use (L)	12,594 ± 208	13,301 ± 176^b^	14,856 ± 179	11,228 ± 154^b^	11,480 ± 136	13,719 ± 183^b^	11,923 ± 170	13,668 ± 178^b^
Acidifying emissions (g SO_2_eq)	4.1 ± 0.1	3.9 ± 0.1^b^	5.2 ± 0.1	3.2 ± 0.1^b^	3.8 ± 0.1	4.4 ± 0.1^b^	4.2 ± 0.1	4.2 ± 0.1
Eutrophying emissions (g PO_4_^3−^eq)	23.6 ± 0.4	21.6 ± 0.3^b^	28.7 ± 0.4	18.4 ± 0.2^b^	21.7 ± 0.3	24.3 ± 0.3^b^	23.3 ± 0.3	23.6 ± 0.3
GHGE (kg CO_2_eq)	10.0 ± 0.3	8.8 ± 0.3^b^	12.4 ± 0.3	7.3 ± 0.2^b^	9.1 ± 0.3	10.1 ± 0.2^b^	9.6 ± 0.3	9.9 ± 0.2
Land use (m^2^)	18.0 ± 0.3	17.1 ± 0.4	22.4 ± 0.4	14.4 ± 0.2^b^	17.3 ± 0.3	18.8 ± 0.3^b^	18.1 ± 0.4	18.5 ± 0.3
Economic	3.0 ± 0.03	3.4 ± 0.03^b^	3.2 ± 0.02	3.2 ± 0.02	2.9 ± 0.03	3.4 ± 0.03^b^	2.9 ± 0.03	3.4 ± 0.02^b^
Share of budget to food (%)	31.6 ± 1.1	18.1 ± 0.4^b^	24.3 ± 0.7	25.9 ± 0.7^b^	32.7 ± 1.0	21.6 ± 0.6^b^	31.4 ± 0.9	21.2 ± 0.6^b^
Sociocultural	3.7 ± 0.02	4.2 ± 0.02^b^	3.9 ± 0.02	4.0 ± 0.01^b^	4.0 ± 0.01	3.9 ± 0.02^b^	4.0 ± 0.02	3.9 ± 0.01^b^
Frequency of meals from a fast-food or pizza place (times during the last 7 days)	2.3 ± 0.05	0.9 ± 0.03^b^	2.0 ± 0.04	1.4 ± 0.03^b^	1.8 ± 0.04	1.6 ± 0.04^b^	1.8 ± 0.05	1.6 ± 0.04^b^
Frequency of ready-to-eat products (times during the last 30 days)	2.3 ± 0.1	1.5 ± 0.1^b^	2.2 ± 0.1	1.8 ± 0.1^b^	2.1 ± 0.1	1.9 ± 0.1	1.8 ± 0.1	2.1 ± 0.1^b^
Frequency of frozen meals and pizza (times during the last 30 days)	3.5 ± 0.2	2.1 ± 0.1^b^	2.7 ± 0.1	2.7 ± 0.1	2.0 ± 0.1	3.0 ± 0.1^b^	2.6 ± 0.1	2.7 ± 0.1

*Abbreviations* NHANES, National Health and Nutrition Examination Survey; SDI-US, sustainable diet index-US; NRF9.3, Nutrient Rich Foods 9.3; MAR, mean nutrient adequacy ratio; GHGE, greenhouse gas emissions.

^a^Estimated mean and standard error were obtained using the linear regression model and weighted (dietary Day 1 sample weights).

^b^Mean values within a row were significantly different between two groups by a two-sample t-test.

Question-5. Does the index correctly measure what it should measure (sustainability of diets)?

Adults in the fifth quintile of SDI-US had significantly higher consumption of whole grains, total vegetables, total fruit, soybean products, and nuts and seeds and lower consumption of refined grains, dairy, meat, solid fats, and added sugar than those in the first quintile. Similarly, both aMed and EAT-Lancet diet scores were significantly higher among adults in the highest SDI-US quintile compared to the lowest quintile (aMed: 4.6 vs. 3.2; EAT-Lancet diet score: 9.9 vs. 8.7, *p* < .0001 for both). These findings suggest that the SDI-US is positively associated with consumption of healthy plant-based foods described in DGA food groups, the aMed score, and the EAT-Lancet diet score (Table 5).

**Table 5:** Weighted mean and standard error of selected food group consumption, alternative Mediterranean diet score, and EAT-Lancet diet score by sustainable diet index-US quintiles, US adults, NHANES 2007–2018

	Weighted mean ± standard error^a^					
	Q1	Q2	Q3	Q4	Q5	p-value^b^
**Food groups in the DGAs**						
Total grains (ounce eq/d)	7.3 ± 0.1	6.8 ± 0.1	6.7 ± 0.1	6.4 ± 0.1	5.7 ± 0.1	< 0.0001
Whole grains (ounce eq/d)	0.6 ± 0.02	0.7 ± 0.02	0.9 ± 0.04	1.0 ± 0.04	1.1 ± 0.04	< 0.0001
Refined grains (ounce eq/d)	6.7 ± 0.1	6.0 ± 0.1	5.8 ± 0.1	5.4 ± 0.1	4.6 ± 0.1	< 0.0001
Total vegetables (cup eq/d)	1.4 ± 0.03	1.5 ± 0.03	1.5 ± 0.02	1.7 ± 0.04	1.8 ± 0.04	< 0.0001
Dark green vegetables (cup eq/d)	0.1 ± 0.01	0.1 ± 0.01	0.1 ± 0.01	0.2 ± 0.01	0.3 ± 0.02	< 0.0001
Total red and orange vegetables (cup eq/d)	0.3 ± 0.01	0.3 ± 0.01	0.4 ± 0.01	0.4 ± 0.01	0.5 ± 0.01	< 0.0001
Total starchy vegetables (cup eq/d)	0.5 ± 0.01	0.5 ± 0.02	0.4 ± 0.01	0.4 ± 0.01	0.4 ± 0.01	< 0.0001
Other vegetables (cup eq/d)	0.5 ± 0.01	0.5 ± 0.02	0.6 ± 0.01	0.6 ± 0.02	0.7 ± 0.02	< 0.0001
Total fruit (cup eq/d)	0.6 ± 0.02	0.8 ± 0.03	0.9 ± 0.03	1.0 ± 0.03	1.3 ± 0.03	< 0.0001
Total dairy (cup eq/d)	1.9 ± 0.04	1.7 ± 0.03	1.6 ± 0.04	1.5 ± 0.03	1.3 ± 0.02	< 0.0001
Total protein foods (ounce eq/d)	7.6 ± 0.1	6.9 ± 0.1	6.2 ± 0.1	5.8 ± 0.1	5.1 ± 0.1	< 0.0001
Total meat, poultry, and seafood (ounce eq/d)	6.4 ± 0.1	5.5 ± 0.1	4.7 ± 0.1	4.3 ± 0.1	3.6 ± 0.1	< 0.0001
Eggs (ounce eq/d)	0.6 ± 0.02	0.6 ± 0.02	0.6 ± 0.02	0.6 ± 0.02	0.5 ± 0.02	0.0068
Soybean products (ounce eq/d)	0.03 ± 0.004	0.1 ± 0.01	0.1 ± 0.01	0.1 ± 0.01	0.1 ± 0.01	< 0.0001
Nuts and seeds (ounce eq/d)	0.6 ± 0.03	0.7 ± 0.1	0.9 ± 0.1	0.8 ± 0.04	0.8 ± 0.04	< 0.0001
Oils (grams/d)	29.7 ± 0.4	27.8 ± 0.6	26.8 ± 0.5	25.2 ± 0.5	23.1 ± 0.4	< 0.0001
Solid fats (grams/d)	49.2 ± 0.7	41.5 ± 0.6	36.7 ± 0.6	33.2 ± 0.5	26.7 ± 0.5	< 0.0001
Added sugar (grams/d)	26.6 ± 0.5	20.4 ± 0.4	16.8 ± 0.3	14.5 ± 0.3	10.6 ± 0.2	< 0.0001
**aMed score**	3.2 ± 0.03	3.5 ± 0.04	3.9 ± 0.04	4.1 ± 0.04	4.6 ± 0.04	< 0.0001
**EAT-Lancet diet score**	8.7 ± 0.03	9.0 ± 0.03	9.3 ± 0.03	9.5 ± 0.04	9.9 ± 0.04	< 0.0001

*Abbreviations* NHANES, National Health and Nutrition Examination Survey; Q, quintile; DGAs, Dietary Guidelines for Americans; aMed, alternative Mediterranean diet score

^a^Estimated mean and standard error were obtained using the linear regression model and weighted (dietary Day 1 sample weights)

^b^*P* values for differences between quintile 1 and quintile 5 were obtained using a two-sample t-test

### Sensitivity analyses

Results from sensitivity analyses comparing modified SDI-US with the original SDI-US are found in Supplementary Table 3. When replacing the NRF9.3 with the HEI-2015, the proportion of adults classified into the same or adjacent quintiles was 99.6% with no complete misclassification (weighted kappa, 0.84; correlation, 0.97). The proportion of perfect agreement between the modified SDI-US using five environmental indicators and the original SDI-US was 95.9% (weighted kappa, 0.98; correlation, 0.999). When adding food security and food price levels to the economic sub-index, the proportion of adults classified into the same or adjacent quintiles was 96.6% with no complete misclassification (weighted kappa, 0.72; correlation, 0.91). When adding eating together with family or friends to the sociocultural sub-index, the proportion of adults classified into the same or adjacent quintiles was 100.0% with no complete misclassification (weighted kappa, 0.91; correlation, 0.99).

## Discussion

The SDI-US reflected the key features of sustainable diets by integrating four sub-indices (nutritional, environmental, economic, and sociocultural) as comparable to the SDI-France. The SDI-US had acceptable content validity, supported by similar correlation coefficients (moderate to high) between each sub-index or the modified index after removing each sub-index and the total SDI-US. The SDI-US showed acceptable construct validity, as participants with a higher SDI-US were more likely to consume healthy plant-based foods and follow the Mediterranean diet and the EAT-Lancet diet. The overall score of the SDI-US was 13 (possible range: 4–20).

Studies have measured sustainable diets using various indicators [35, 36], such as, GHGE, land use, consumption of animal source foods, and diet quality [35]. However, there is an inevitable heterogeneity in methodologies [35, 36] and a single indicator does not operationalize the multidimensionality of sustainable diets [36]. To date, several composite indices using multiple indicators have been developed: SDI-France [5], SDI-Spain [8], EAT-Lancet score [28], planetary health diet index [37, 38], EAT-Lancet index [39], Dietary Index [40], and Healthy Reference Diet score [41]. The latter five indices, which only use data on food consumption, assess adherence to the EAT-Lancet diet [28, 37–41]. As a result, they neglect other important environmental, economic, and sociocultural dimensions of diets. The SDI-France quantifies adherence to sustainable diets with a multidimensional approach [5]. It uses not only dietary consumption data but also food expenditures and dietary practices related to ready-made products, in order to consider all four dimensions of diets [5]. The SDI-US is created using US data based on the scoring system of the SDI-France. Thus, it can be a useful tool to assess the sustainability of the US diet in a holistic manner.

The accuracy of the environmental impacts of foods may vary depending on the database utilized. Although the database of Food Impacts on the Environment for Linking to Diets (dataFIELD) has been widely used to estimate the environmental impacts of US diets, it can only estimate the GHGE and energy demand of US diets [42]. We used a database by Poore and Nemecek to further consider other important environmental impacts (water footprint, nitrogen footprint, and land use). However, both databases are based on meta-analyses of multiple previous studies, and substantial variability in environmental impact values between studies is inevitable (Supplementary Table 4). In addition, in our study, about 40% of reported foods were matched to estimate their environmental impacts. Most of the 60% of reported foods that could not be matched to estimate their environmental impacts were mixed foods or processed foods. Nevertheless, GHGE values (kg CO_2_eq) were comparable between our study and previous studies using dataFIELD. For example, the estimated GHGE in our study was 4.39 in 2007–2008 and 3.68 in 2017–2018 compared to 3.6 in 2005–2010 published by Heller et al. and 2.45 in 2018 published by Bassi and colleagues [42, 43]. Further studies are needed to verify the accuracy of the environmental impacts of individual diets.

The present study has several strengths. First, to our knowledge, this study is the first attempt to assess the degree of multidimensional dietary sustainability in US adults by including nutritional, environmental, economic, and sociocultural of sustainable diets as suggested by the FAO. Second, NHANES is the only survey that provides the range of data needed for the calculation of the total SDI-US. Third, compared to the previous environmental impact factor analysis [42, 43], the SDI-US assesses the expanded range of environmental impacts including water footprint and nitrogen footprint [26].

This study also has some limitations. First, the SDI-US uses only individual data captured through the NHANES surveys, which does not include the following societal dimensions mentioned in the FAO definition: sociocultural factors such as wellbeing (e.g., child labor, animal health and welfare), resilience (e.g., country’s vulnerability to climate change, food production diversity), and food safety (e.g., foodborne disease burden) [44]. Second, nutritional and environmental impact scores derived from a single self-reported 24-hour dietary recall are subject to measurement error and underreporting [20, 21, 45, 46] but sufficient for estimating the mean intake of a population [47]. Such bias may be minimized because of the use of validated instruments and standardized protocols during data collection in NHANES [48, 49] and the use of energy-adjusted nutrient intake [18, 21]. Other sources of measurement error include the nutritional sub-index, which does not take into account how nutrient composition can change during cooking, processing, or storage, and the environmental sub-index which does not account for effects due to cooking, consumer losses, and the impact of other types of environmental pollution (e.g., agrochemicals, antibiotic use, and particulate matter). Third, the metric of the SD-US is an arbitrary function of the scoring chosen for the indices. For example, the score implicitly assumes that a 1-point difference in one sub-index is assumed to be equivalent to a 1-point difference in another sub-index due to equal weighting. Although equal weighting is consistent with the FAO definition of sustainability [1] and has been frequently used in the literature [5–8], arguably other weighting schemes could be used that reflect various programmatic goals and values. Future studies may help clarify what a 1-point difference in indices means in terms of health and environmental impact. In the meantime, describing differences relative to the standard deviation might be helpful. Fourth, although we used similar methods of validity assessment as in other studies [5, 34], these are neither a gold standard nor objective criteria. Finally, since we focused on US adults, our findings may not be generalizable to other populations (e.g., different races/ethnicities, adolescents).

In conclusion, the SDI-US can be used in the US to comprehensively assess alignment with sustainable diets based on a multicriteria approach. Despite growing interest in sustainable diets, overall SDI-US was 13 out of 20 in a nationally representative sample of US adults. Although an accurate method for interpreting the SDI-US scores remains to be determined, there were significant differences between subgroups of the population suggesting disparities. These findings provide a benchmark for the sustainability of the US diet and show that there is an opportunity for improvement.

### Electronic supplementary material

Below is the link to the electronic supplementary material.

Supplementary Material 1

## Data Availability

All data are publicly available at https://wwwn.cdc.gov/nchs/nhanes/Default.aspx.
