# Supplementary material for: Development of a sustainable diet index in US adults
Source: Nutr J. 2024 Apr 10;23:46. doi: 10.1186/s12937-024-00943-3 (PMC11040758; doi:10.1186/s12937-024-00943-3)
Supplement: Supplementary file 1 — Supplementary Material 1 [file 12937_2024_943_MOESM1_ESM.pdf]

Online supplementary material

This appendix is a part of the original submission.

Supplement to: Jung S, Young HA, Simmens SJ, Braffett BH, and Ogden CL. Development of a sustainable diet index in US adults

Supplementary Figure 1. Study participants flow chart, National Health and Nutrition Examination Survey (NHANES) adults aged 20 years and older, 2007–2018

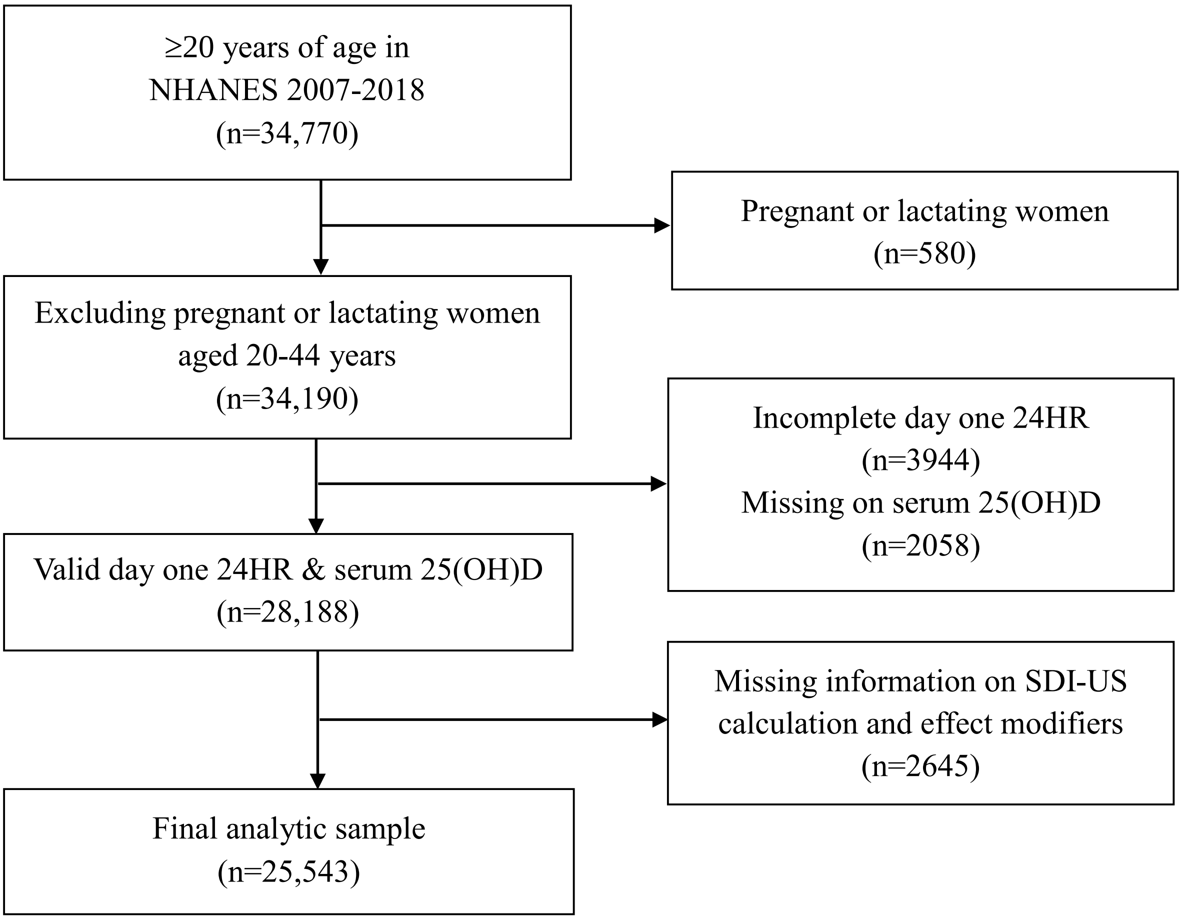

Supplementary Information A. Comparison between the sustainable diet index-France (SDI-France) and the sustainable diet index-US (SDI-US)

Differences between the SDI-France and SDI-US are summarized in **Supplementary Table 1**. Briefly, the main differences are in 1) the number of indicators (7 for the SDI-France vs. 12 for the SDI-US with an expanded number of environmental and sociocultural indicators); and 2) the lack of assessment of some measures (e.g., places of food purchase and dietary energy intake) due to data quality or availability.

Specifically, while the SDI-France used the PANDiet index to both assess the probability that 24 nutrient intakes met the required level (adequacy) and the probability that 24 nutrient intakes did not exceed the recommended level (moderate) [1], the SDI-US used two indicators, Nutrient-Rich Foods (NRF) 9.3 index and mean nutrient adequacy ratio (MAR), to better represent the dietary diversity and nutrient deficiency level of US adults. SDI-US environmental indicators, included water footprint and nitrogen footprint of foods calculated from a comprehensive database based on a meta-analysis of 1530 publications [2], in addition to carbon footprint and land use. And, SDI-US sociocultural indicators included more specific food practice indicators related to ready-to-eat products use instead of just one indicator in the French original version [1]. There is no need for adaptation for the economic indicator (share of food budget).

Measures related to dietary energy intake were not included in the SDI-US due to substantial underreporting issues in any self-reported dietary assessment method, including the 24-hour dietary recall [3]. Unlike other nutrients, underreporting issues may be more critical in total energy intake. Since total energy intake is contained in almost every food and beverage, summing errors in each individual food and beverage would lead to errors in total energy intake to a greater extent than for other nutrients. Thus, it is recommended not to use self-reported energy intake as a true energy intake, but it can be used for adjustment purposes [4]. In addition, SDI-US did not include a measure of places of food purchase since this information is only available at the individual food level and thus cannot be combined like a total intake value (e.g., if someone ate 6 foods a day at different places, this person could have 6 different places where foods were obtained).

References for **Supplementary Information A**

[1] Seconda L., Baudry J., Pointereau P., Lacour C., Langevin B., Hercberg S., et al., Development and validation of an individual sustainable diet index in the NutriNet-Santé

study cohort, Br J Nutr 121 (10) (2019) 1166-1177.
[2] Poore J., Nemecek T., Reducing food's environmental impacts through producers and consumers, Science 360 (6392) (2018) 987-992.
[3] Archer E., Hand G. A., Blair S. N., Validity of U.S. nutritional surveillance:National Health and Nutrition Examination Survey caloric energy intake data, 1971-2010, PLoS One 8 (10) (2013) e76632.
[4] Poslusna K., Ruprich J., de Vries J. H., Jakubikova M., van't Veer P., Misreporting of energy and micronutrient intake estimated by food records and 24 hour recalls, control and adjustment methods in practice, Br J Nutr 101 Suppl 2 (2009) S73-85.

Supplementary Table 1. Comparison between the sustainable diet index-France and the sustainable diet index-US

		SDI-France[a]		SDI-US	
Sub-index	Measure[a]	Included (Y/N)	Indicator	Included (Y/N)	Indicator
Nutritional	Dietary energy balance	Y	Absolute value of difference between energy content needed and intake	N: weak data[d]	N/A
	Dietary energy density	Y	PANDiet score[b]	N: weak data[d]	N/A
	Dietary diversity index			Y	Nutrient-Rich Foods9.3 Index[e]
	Micronutrient deficiencies of vitamins and minerals			Y	Mean Nutrient Adequacy Ratio[f]
Environmental	Water footprint	N: no data	N/A	Y	Freshwater withdrawals (L) per serving food[g]<br>Stress-weighted water use (L) per serving food[g]
	Nitrogen footprint	N: no data	N/A	Y	Acidifying emissions (g SO_2eq, CML2 baseline) per serving food[g]<br>Eutrophying emissions (g PO_4^{3-}eq, CML2 Baseline) per serving food[g]
	Carbon footprint	Y	Partial ReCiPe score[c]	Y	Greenhouse gas emissions (kg CO_2eq, IPCC 2013 includes feedbacks) per serving food[g]
	Non-renewable energy	Y		N: no data	N/A
	Land use	Y		Y	Land use (m^2) per serving food[g]
	Preservation of biodiversity farm	Y	Contribution of organic food to diet	N: no data	N/A
Economic	Affordability	Y	Proportion of income devoted to diet	Y	Money spent foods or eating at the family level / Monthly family income
Sociocultural	Local/regional foods	Y	Places of food purchases	N: no data	N/A
	Diversity of food supply chain	Y		N: no data	N/A
	Ready-made products	Y	Ready-made products	Y	Frequency of meals not home prepared and from a fast-food or pizza place<br>Frequency of ready-to-eat products<br>Frequency of frozen meals/pizza

Abbreviation: SDI, sustainable diet index.

[a] Source: Seconda L, Baudry J, Pointereau P, et al. Development and validation of an individual sustainable diet index in the NutriNet-Santé study cohort. *Br J Nutr* 2019;121:1166-77.

[b] Source: Verger EO, Mariotti F, Holmes BA, Paineau D, Huneau JF. Evaluation of a diet quality index based on the probability of adequate nutrient intake (PANDiet) using national French and US dietary surveys. *PLoS One* 2012;7:e42155.

[c] Source: Kramer GF, Tyszler M, Veer PV, Blonk H. Decreasing the overall environmental impact of the Dutch diet: how to find healthy and sustainable diets with limited changes. *Public Health Nutr* 2017;20:1699-709.
[d] "weak data" indicates that data is available, but its quality is not sufficient to use (underreporting of dietary energy intake is a major challenge in any self-reported dietary assessment method).
[e] Sources: Fulgoni VL, 3rd, Keast DR, Drewnowski A. Development and validation of the nutrient-rich foods index: a tool to measure nutritional quality of foods. *J Nutr* 2009;139:1549-54; Drewnowski A. Defining nutrient density: development and validation of the nutrient rich foods index. *J Am Coll Nutr* 2009;28:421s-6s.
[f] Source: Guthrie HA, Scheer JC. Nutritional adequacy of self-selected diets that satisfy the four food groups guide. *J Nutr Education* 1981;13:46-9.
[g] Source: Poore J, Nemecek T. 2018. Reducing food's environmental impacts through producers and consumers. Science 360:987-992 and Bryan T, Hicks A, Barrett B, et al. An environmental impact calculator for 24-h diet recalls. *Sustainability* 2019;11(23):6866

Supplementary Table 2. Demographic characteristics by NHANES cycle, US adults, NHANES 2007–2018 (n=25,543)

	No. of participants (weighted %)[a]					
	2007–2008 (n=3996)	2009–2010 (n=4887)	2011–2012 (n=4120)	2013–2014 (n=4408)	2015–2016 (n=4212)	2017–2018 (n=3920)
Age group, y						
20–39	1230 (36.1)	1581 (35.6)	1466 (35.6)	1470 (34.7)	1374 (34.7)	1143 (35.1)
40–59	1278 (39.4)	1687 (39.6)	1380 (38.6)	1531 (37.5)	1437 (37.5)	1266 (35.2)
≥60	1488 (24.6)	1619 (24.8)	1274 (25.8)	1407 (27.7)	1401 (27.8)	1511 (29.7)
Sex						
Male	1988 (47.3)	2414 (48.9)	2079 (49.6)	2140 (49.6)	2075 (49.0)	1944 (49.1)
Female	2008 (52.7)	2473 (51.1)	2041 (50.4)	2268 (50.4)	2137 (51.0)	1976 (50.9)
Race/Hispanic origin						
Hispanic	1084 (12.7)	1304 (12.5)	797 (13.8)	944 (14.2)	1277 (14.4)	843 (14.9)
Non-Hispanic white	2006 (72.5)	2503 (71.3)	1660 (68.2)	2024 (67.0)	1505 (66.4)	1498 (64.2)
Non-Hispanic black	763 (10.0)	831 (10.5)	1036 (10.7)	854 (10.7)	854 (10.1)	867 (10.4)
Other[b]	143 (4.7)	249 (5.8)	627 (7.4)	586 (8.1)	576 (9.1)	712 (10.5)
Education level						
Less than high school graduate	1211 (19.6)	1338 (18.0)	889 (15.0)	863 (14.3)	922 (12.8)	700 (10.1)
High school graduate or GED	961 (25.1)	1115 (22.4)	862 (19.8)	1000 (22.3)	947 (21.4)	963 (28.9)
Some college or above	1824 (55.4)	2434 (59.6)	2369 (65.2)	2545 (63.4)	2343 (65.8)	2257 (61.0)
Household size						
Single-person household	582 (14.1)	710 (14.3)	607 (13.4)	633 (14.2)	614 (14.2)	582 (12.9)
Multi-person household	3414 (85.9)	4177 (85.7)	3513 (86.6)	3775 (85.8)	3598 (85.8)	3338 (87.1)

Abbreviations: NHANES, National Health and Nutrition Examination Survey; GED, general equivalency diploma.

[a] Sample sizes are unweighted and all other estimates are weighted (dietary day 1 weights).

[b] "Other" includes race/Hispanic origin other than non-Hispanic white, non-Hispanic black, and Hispanic, including multiracial.

Supplementary Table 3. Comparison of the sustainable diet index-US and modified sustainable diet index-US, US adults, NHANES 2007–2018

	Median (range)	Agreement with the original SDI-US (%) [a]					Weighted kappa [a]	Correlation [b]
		Same group (S)	Adjacent group (A)	Opposite group (O)	Others	S+A		
**Original SDI-US**	13.2 (4.3–20.0)	–	–	–	–	–	–	–
**Modified version**								
mSDI-US-1 NRF9.3 → HEI-2015	13.2 (4.5–20.0)	75.3	24.3	0.0	0.4	99.6	0.84	0.97
mSDI-US-2 Excluded freshwater use	13.2 (4.3–20.0)	95.9	4.1	0.0	0.0	100.0	0.98	0.999
mSDI-US-3 Included food security and food price level	13.7 (5.2–20.0)	58.3	38.3	0.0	3.4	96.6	0.72	0.91
mSDI-US-4 Included eating together	12.9 (4.3–20.0)	85.1	14.9	0.0	0.0	100.0	0.91	0.99

Abbreviations: NHANES, National Health and Nutrition Examination Survey; CI, confidence interval; SDI-US, sustainable diet index-US; mSDI-US, modified sustainable diet index-US; NRF9.3, Nutrient-Rich Foods9.3; HEI, Healthy Eating Index.
Note: mSDI-US-1 was calculated using the Healthy Eating Index-2015 instead of using Nutrient-Rich Foods9.3 index because better nutritional composition does not necessarily translate into an overall healthier diet; mSDI-US-2 was calculated using 5 environmental indicators after excluding one water-related indicator (freshwater use) to consider the possible effect of double counting on water in relation to the other indicators; mSDI-US-3 was calculated by additionally including food security and food price levels to the economic sub-index to better represent food affordability using NHANES 2011–2018 due to data availability; mSDI-US-4 was calculated by additionally including eating together with family or friends to the sociocultural sub-index because higher frequency of ready-to-eat meals may not necessarily indicate negative sociocultural practices related to social exchange or trying diverse recipes if people are eating together using NHANES 2007–2010 due to data availability.

[a] The proportions of agreement and the weighted kappa coefficients were obtained from a cross-classification analysis to evaluate the agreement of classification into quintiles between the original SDI-US and the modified SDI-US. The correct classification indicated that participants were classified into the same quintile groups (e.g., original SDI-US quintile 5 and the modified SDI-US quintile 5), and the gross misclassification indicated that participants were classified into the opposite quintile groups (e.g., original SDI-US quintile 5 but the modified SDI-US quintile 1).
[b] The Pearson correlation coefficients of the original SDI-US with the modified SDI-US were estimated using the linear regression model.

Supplementary Table 4. Greenhouse gas emission (kg CO_2eq per 1 kg) between Poore and Nemecek database and dataFIELD

	Poore & Nemecek [a]	dataFIELD [b]
Wheat & rye	1.57	0.36
Barley	1.17	0.39
Oatmeal	0.41	0.50
Rice	1.40	1.54
Potatoes	0.46	0.22
Cane sugar	3.20	0.70
Beet sugar	1.81	0.39
Pulses	0.62	0.26
Peas	0.31	0.70
Nuts	0.43	0.99
Soymilk	0.91	0.26
Palm oil	7.96	7.22
Sunflower oil	3.75	2.65
Rapeseed oil	4.19	2.28
Olive oil	5.96	3.21
Tomatoes	2.09	0.47
Onions	0.50	0.27
Citrus fruit	0.39	0.40
Bananas	0.86	0.37
Apples	0.43	0.23
Grapes	1.53	0.48
Wine	1.81	0.78
Coffee	1.71	6.28
Beef	99.48	45.69
Lamb	39.72	34.75
Pork	12.31	5.56
Poultry	9.87	4.19
Milk	3.06	11.02
Eggs	4.67	3.75
Fish	13.63	5.72
Crustaceans	26.87	30.66

Abbreviation: dataFIELD, database of Food Impacts on the Environment for Linking to Diets.
[a] Poore J, Nemecek T. 2018. Reducing food's environmental impacts through producers and consumers. Science 360:987-992.
[b] dataFIELD is available at http://css.umich.edu/page/datafield.
